# Spontaneous recovery from overexpectation in an insect

**DOI:** 10.1038/s41598-022-13800-2

**Published:** 2022-06-14

**Authors:** Kanta Terao, Yukihisa Matsumoto, Beatriz Álvarez, Makoto Mizunami

**Affiliations:** 1 College of Liberal Arts and Sciences, Tokyo Medical and Dental University, Ichikawa, 272-0827 Japan; 2 Arts and Sciences, Research Institute for Letters, Waseda University, 1-24-1, Toyama, Shinjuku 162-8644 Japan; 3 Facultad de Ciencias de La Salud, Universidad Pública de Navarra, 31006 Pamplona, Spain; 4 Faculty of Science, Hokkaido University, Sapporo, 060-0810 Japan

**Keywords:** Neuroscience, Psychology

## Abstract

In associative learning in mammals, it is widely accepted that learning is determined by the prediction error, i.e., the error between the actual reward and the reward predicted by the animal. However, it is unclear whether error-based learning theories are applicable to the learning occurring in other non-mammalian species. Here, we examined whether overexpectation, a phenomenon that supports error-based learning theories, occurs in crickets. Crickets were independently trained with two different conditioned stimuli (CSs), an odour and a visual pattern, that were followed by an appetitive unconditioned stimulus (US). Then the two CSs were presented simultaneously as a compound, followed by the same US. This treatment resulted in a reduced conditioned response to the odour CS when tested immediately after training. However, the response to the CS was partially recovered after 1 day. These results are the first to show overexpectation and its spontaneous recovery in an invertebrate species. While the results showing overexpectation are in agreement with the prediction by the Rescorla-Wagner model, a major form of error-based learning theories, the ones showing spontaneous recovery are not. Our results suggest that conventional error-based learning models account for some, but not for all essential features of Pavlovian conditioning in crickets.

## Introduction

Animals collect information from their surroundings, assess its reliability, and integrate it to optimise their behaviour in a constantly changing environment. Pavlovian (classical) conditioning is a basic form of associative learning that allows animals to establish certain predictions about their environment and that takes place when a neutral cue (conditioned stimulus, CS) is paired with a biologically relevant event (unconditioned stimulus, US). This type of learning has been found in many taxa of vertebrate and invertebrate animals^1^, but whether basic computational rules underlying Pavlovian conditioning are conserved among different species remains largely unknown.

Modern theories of Pavlovian conditioning in mammals, known as error-based learning theories, assume that conditioning occurs because of the discrepancy (i.e., error) between the reward that the animal actually receives and the reward it predicted based on its previous experience. Among these theories, the most influential ones are the Rescorla-Wagner model^2^ and the attentional theories proposed by Mackintosh^3^ and by Pearce and Hall^4^ (Table 1). These theories were proposed after the finding of blocking^5^, a phenomenon that did not support the notion of temporal contiguity^6^ or contingency^7^ since it showed that the mere pairing of two events or the relative probability of occurrence of the US in the presence of CS compared to the occurrence of the US in the absence of the CS was not sufficient for conditioning to be effective^8^. In the original study on blocking conducted by Kamin^5^, animals in the control group were exposed to paired presentations of a compound of two CSs (A and B) followed by a US (AB+), whereas animals in the experimental group received the same training (AB+) but after having been previously trained with one of the CSs (A +). Thus, for animals in the experimental group, the compound was composed of a novel CS (B) and an already known CS (A). When tested for their response to B, animals in the experimental group responded significantly less than did animals in the control group. This observation was inconsistent with what would be expected if either temporal contiguity or contingency principles applied: both groups should have shown the same levels of response to B since it had always been followed by a reward in AB + training. Instead, error-based learning theories are able to account for the phenomenon. For example, the Rescorla-Wagner model predicts that no learning for B should occur in AB+ training since the occurrence of the US is fully predicted by A and hence there is no prediction error in AB+ training.

**Table 1:** Learning theories to account for blocking.

Theory	Equation
a. Rescorla-Wagner model	ΔV = α(λ − V_Σ_) V_Σ_ = V_A_ + V_B_ + ··· + V_X_
b. Attentional theory by Mackintosh	ΔV = α_A_(λ − V_A_) α_A_ is increased if |λ − V_A_| <|λ − V_X_| α_A_ is decreased if |λ − V_A_| ≧ |λ − V_X_|
c. Attentional theory by Pearce and Hall	ΔV_A_^n^ = S_A_ α_A_^n^ λ α_A_^n^ =|λ^n-1^ − V_Σ_^n-1^|

V: associative strength, which corresponds to US prediction; V_A_, V_B_ and V_X_: associative strength for stimuli A, B and X, respectively; ΔV: change in associative strength in a particular trial; α: learning-rate parameter reflecting attentional process (0 < α < 1); α_A_^n^: learning-rate parameter for stimulus A in trial n; λ: maximum strength that the CS-US association can achieve, set by the magnitude of the US; S: salience, which corresponds to intensity of the stimulus. Description of the equations follows Pearce and Hall^4^ with little modification.

We recently obtained evidence suggesting that error-based learning theories account for Pavlovian conditioning in crickets, for the first time in an invertebrate^9–11^. We showed blocking with both appetitive and aversive procedures, we obtained evidence of one-trial blocking, a special case of blocking, and we also performed pharmacological analysis of a learning phenomenon named “auto-blocking”^9,10^. Based on these results, we suggested the Rescorla-Wagner model provides the best accounts for Pavlovian conditioning in crickets^9^, and we provided evidence that error-based learning theories are applicable to this insect. On the other hand, a report of blocking in honeybees did not fully support the Rescorla-Wagner model^12^, and we therefore wanted to obtain more evidence to examine the validity of the model.

**Figure 1:**
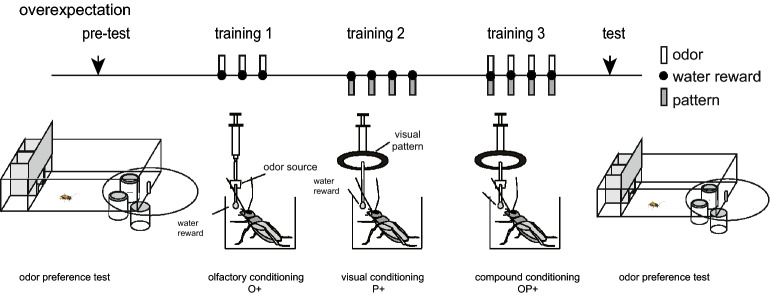
Experimental procedure for overexpectation in olfactory conditioning in crickets. A group of water-deprived crickets, individually placed in a beaker, was subjected to pairings of an odour CS with water US during training 1, to pairings of a visual pattern CS with water US during training 2, and to pairings of an odour and a visual pattern compound with water US during training 3. The relative preference for the conditioned odour and a control odour was tested before and after training in a test chamber.

Overexpectation is a learning phenomenon predicted by the Rescorla-Wagner model. In a standard demonstration of overexpectation^13^ (Fig. 1), experimental and control groups are first trained with two different stimuli, A and B, followed by the same reward (US) (A+ and B+ training), until they achieve asymptotic conditioning. Then, in a second phase, the experimental group is exposed again to stimuli A and B presented in compound followed by the same US (AB+ training). In rodents, this training results in less CR to A in the experimental group than in the control group^13^. The results are explained in terms of the Rescorla-Wagner model: AB+ training should lead to prediction of twice the amount of US due to preceding A+ and B+ training, but the subject only receives once the amount of US. Consequently, negative prediction error occurs, and this produces a reduction of excitatory associations for A (Table 2). Thus, when overexpectation is found, it is regarded as a phenomenon that supports the error-based learning theories^14^. However, as Rescorla himself noted, spontaneous recovery occurs after overexpectation in rats^15^. Such an increase in the response to A indicates that the initial excitatory associations of A and B with the reward are not reduced by overexpectation training, and hence the Rescorla-Wagner model needs to be revised so that it can account for overexpectation and its spontaneous recovery.

**Table 2:** Prediction based on the Rescorla and Wagner model for overexpectation.

	Actual reward	Predicted reward for A at the beginning of training	Predicted reward for B at the beginning of training	Prediction error
Pre-test	0	0	0	0
Training 1 (A+)	1	0	–	1 = 1–0
Training 2 (B+)	1	–	0	1 = 1–0
Training 3 (AB+)	1	1	1	− 1 = 1 − (1 + 1)
Test	–	Less than 1 More than 0	–	–

To simplify the predictions, we assume that λ = 1 and that each of the trainings provides a sufficient effect to achieve conditioning.

Overexpectation has been demonstrated in several species of birds and mammals, including humans in appetitive conditioning^14,16,17^ but, to the best of our knowledge, overexpectation and its spontaneous recovery have not been reported in any invertebrate species.

Here we investigated whether overexpectation and its spontaneous recovery occurs in crickets and we also investigated whether Pavlovian conditioning in insects can be accounted for by the error-based learning theories.

## Results

### Experiment 1

**Figure 2:**
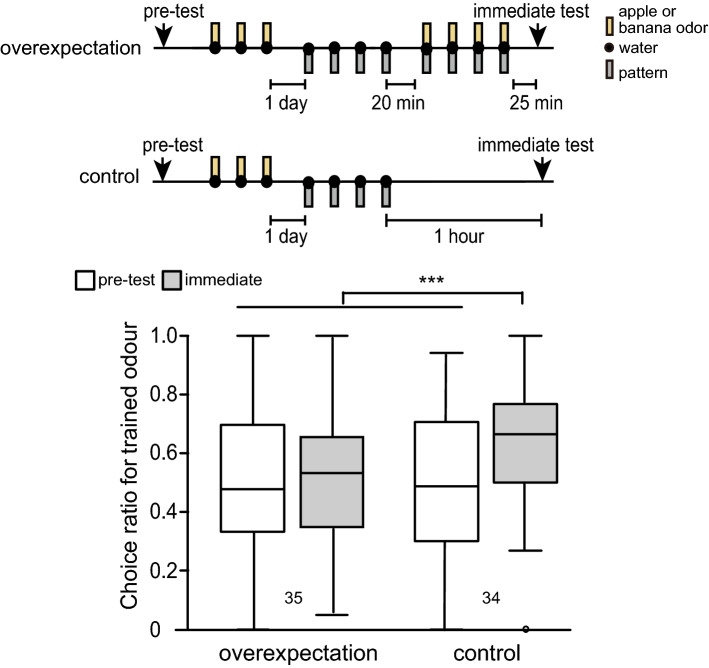
Results of Experiment 1: Overexpectation of olfactory learning in crickets. Crickets in the overexpectation group (n = 35) were subjected to 3 conditioning trials in which an odour was paired with water (O+ training). To counterbalance for the odours used, half of the crickets were trained with either apple or banana odour as the CS. On the next day, they were subjected to 4 conditioning trials in which a white-centre and black-surrounding visual pattern was paired with water (P+ training). After 20 min, they were subjected to 4 odour-visual pattern compound conditioning trails (OP+ training). Crickets in the control group (n = 34) were subjected to 3 O+ training trials and 1 day later they were subjected to 4 P+ training trials. The ITI was 5 min. Relative odour preference was tested before training (pre-test) and immediately after training (immediate test). The latter is 1 h after completion of P+ training in both groups, which matches to 25 min after completion of OP+ training in the overexpectation group. The results before (white) and immediately after (light grey) training are shown as box and whisker diagrams. A GLMM was used to examine relative preferences for the conditioned odour (Supplemental Table 1). Statistical significance is shown as asterisks (***p < 0.001).

To demonstrate overexpectation in crickets, we used the following procedure (Fig. 2): During the first training phase of the experiment (training 1), crickets received three O+ olfactory conditioning trials. To counterbalance the stimuli employed, apple odour was used as the CS for half of the animals and banana odour was used for the other half. Then, in a second phase (training 2) they received four P+ visual pattern conditioning trials. After this training, crickets in the control group were left undisturbed, whereas crickets in the overexpectation group received four OP+ compound conditioning trials (training 3). The odour and pattern used in the OP+ trials were the same as those used during training 1 and training 2, respectively. The relative preference between apple and banana odours was tested before the experiment started (pre-test) and immediately after conditioning of the visual pattern (immediate test), i.e., 1 h after training 2 for both groups and 25 min after training 3 for the overexpectation group. We previously showed that a 25-min rest period is sufficient to establish anaesthesia-resistant memory and hence the level of memory 25 min after training is as high as that 1 h after training^18^.

In pre-test, crickets in the overexpectation and control groups did not differ in their preference for stimulus O (i.e., the odour) (Fig. 2; training term, *p* = 0.196, z = 1.29, see Supplemental Table 1). In immediate test, crickets in the control group exhibited a significantly increased preference for stimulus O and the preference was greater than that in the overexpectation group (interaction term, *p* < 0.001, z = 4.96). The preference in overexpectation group was not significantly increased after training compared to that before training (test term, *p* > 0.05, z = − 0.524). These results are consistent with the view that the preference for O, which increased after O + and P + training, was reduced by subsequent OP+ trainings, thereby supporting the occurrence of overexpectation in crickets (please see “Discussion”).

### Experiment 2

**Figure 3:**
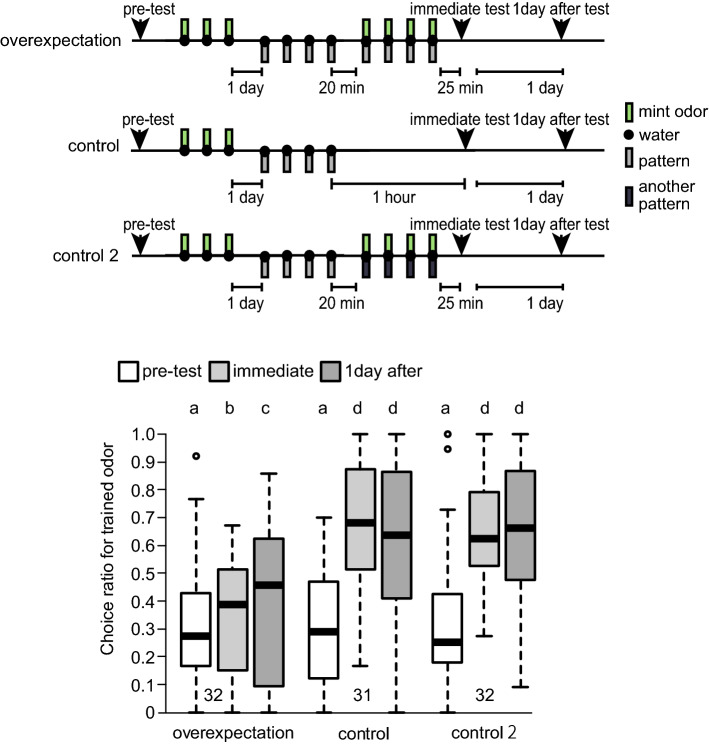
Results of Experiment 2: Effect of overexpectation lasts 1 day. Crickets in one group (overexpectation group, n = 32) were subjected to 3 conditioning trials in which mint odour was paired with water (O+ training). One day later, they were subjected to 4 conditioning trials in which a white-centre and black-surrounding pattern was paired with water (P+ training), and 20 min later, they were subjected to 4-trial pairing of an odour-pattern compound with water (OP+ training). Crickets in the control group (n = 31) were subjected to 3-trial O+ training and 1 day later they were subjected to 4-trial P+ training. The ITI was 5 min. Crickets in the control 2 group (n = 32) were subjected to 3 O+ training trials. After 1 day, they were subjected to 4 P+ training trials and then 4 pairings of a compound consisting of mint odour and black-centre and white-surrounding pattern followed by water (OP_2_+ training). Relative odour preference was tested before training (pre-test), 1 h after completion of P+ training (immediate test), and 1 day after conditioning. The results in pre-test (white), immediate test (light grey) and 1 day after test (dark grey) are shown as box and whisker diagrams. The horizontal line in the box is the median and the box represents the 25–75 percentiles in this and in all following figures. Whiskers extend to extreme values as long as they are within a range of 1.5 * box length. The outliers are shown as open circles. A GLMM was used to examine relative preferences for the conditioned odour (Supplemental Table 2 and 3). Different letters indicate significant difference in pairwise comparisons from post-hoc tests (*p* < 0.05).

We then tested whether the effect of overexpectation could last longer (Fig. 3). To clarify the robustness of overexpectation, a different set of odours, mint/vanilla odours, was used. Before conditioning, the relative preference for mint/vanilla odours was tested. Crickets in both the overexpectation and control groups received three mint O+ conditioning trials. Then they received four P+ conditioning trials. As in Experiment 1, after this training, crickets in the control group were left undisturbed, whereas crickets in the overexpectation group received four OP+ conditioning trials. The relative preference for mint and vanilla odours was tested twice: 1 h after P+ training and 1 day after conditioning.

In Experiment 1, the overexpectation and control groups differed in the total amounts of exposure to the CSs and to the US: The reduced response of the overexpectation group might be due to larger number of exposure to the CS and the US. Thus, we added another control group, hereafter referred to as control 2, that received the same amount of stimulation as that in the overexpectation group. Like crickets in the other two groups, they received three mint O+ conditioning trials and four P+ conditioning trials. After this training, they received four OP_2_+ conditioning trials, in which P_2_ refers to a different visual pattern (BW) from the one used for the other two groups (WB). The odour used during training 3 (i.e., compound conditioning trials) in the control 2 group was the same as that used during training 1, i.e., the training received by control 2 was the same as the training that would be typically used for the study of blocking. The relative preference for mint and vanilla odours was tested before training, 1 h after P+ training and 1 day after conditioning.

In pre-test, crickets in the overexpectation, control and control 2 groups did not show any differences in their preference for stimulus O (Fig. 3; *p* = 1, z = − 0.930, 0.270 and 0.423, supplemental Tables 2 and 3). After training, all groups showed a significantly higher preference for O in the immediate and 1-day-after tests (*p* < 0.05, z < − 2.26). The preference in the overexpectation group was significantly lower than the preference in the control and control 2 groups when tested both immediately after training and 1 day after the first test (*p* < 0.001, z < − 6.01). The preference for stimulus O in both the control and control 2 groups was not significantly different between the immediate and 1-day-after tests (*p* > 0.370, z > 0.420), but significant differences were found between the pre and immediate test and between before training and 1-day-after test (*p* < 0.001, z < − 13.0). In contrast, within the overexpectation group, crickets´ preference for stimulus O was significantly higher when tested 1 day after training than when tested immediately after training (*p* < 0.05, z < − 2.25). These results show spontaneous recovery from overexpectation.

### Experiment 3

**Figure 4:**
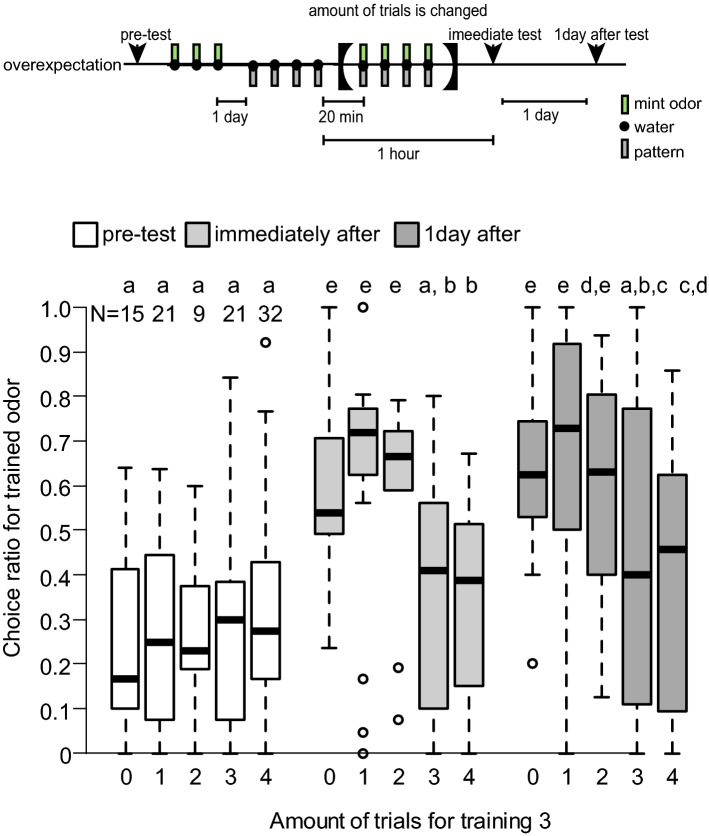
Results of Experiment 3: Effect of overexpectation depends on the number of compound conditioning trials. Crickets were subjected to 3 conditioning trials in which mint odour was paired with water (O+ training). One day later, they were subjected to 4-trial pairing of a white-centre and black-surrounding pattern with water (P+ training). Twenty min later, they were subjected to 0 to 4 pairings of an odour-pattern compound with water (OP+ training). The relative odour preference was tested before training, 1 h after completion of P+ training, and 1 day after conditioning. The results in pre-test (white), immediate test (light grey) and 1 day after test (dark grey) are shown as box and whisker diagrams. A GLMM was used to examine relative preferences for the conditioned odour (Supplemental Tables 4 and 5). Different letters indicate significant difference in pairwise comparisons from post-hoc tests (*p* < 0.05).

We conducted another experiment to test whether the effect of overexpectation depends on the number of compound conditioning trials conducted during training 3. We used the following procedure (Fig. 4): After receiving three mint O+ conditioning trials and four P+ conditioning trials, crickets received zero to four OP+ conditioning trials. The relative preference between mint and vanilla odours was tested 1 h after P+ training and 1 day after conditioning.

In pre-test, no differences were found between the groups in preference for stimulus O (Fig. 4; *p* = 1, z < 0.570; see Supplemental Tables 4 and 5). After training, crickets showed a significantly higher preference for O both in the immediate test and in the 1-day-after test (*p* < 10^–42^, 13.0 < z < 15.0), but such preference was found to become lower depending on the number of OP+ trials received during training 3 (*p* < 0.001, − 9.96 < z < 5.64). Specifically, the preference of animals that received 4 OP+ trials during training 3 was significantly higher in the 1-day-after test than in the pre-test and in immediate test (*p* < 0.001, z < − 3.00). The preference in immediate test was significantly higher than that in pre-test, too (*p* < 0.05, z = − 2.03). In immediate test and 1-day-after test, animals that received 4 OP+ trials showed significantly lower preference than animals that received 0, 1 and 2 OP+ trials (*p* < 0.05, z > 2.50). In pre-test, the preference of animals that received 4 OP+ trials were not significantly different from that of animals that received 0, 1, 2 and 3 OP+ training trials (*p* = 1, − 1.15 < z < 0.30). On the contrary, the preference of animals that received 3 OP+ trials was not significantly different between the pre-test, the immediate test and the 1-day-after test (*p* > 0.10, z < − 0.9), though, in immediate test and 1-day-after test, animals that received 3 OP+ trials showed significantly lower preference than animals that received 0, 1 and 2 OP+ trials (*p* < 0.05, z > 2.60). In the pre-test, the preference of animals that received 3 OP+ trials was not significantly different from that of animals that received 0, 1 or 2 OP+ trials (*p* = 1, − 1.40 < z < − 0.30). Overexpectation occurs if animals received 3 or 4 OP+ trials, and spontaneous recovery is seen if animals received 4 OP+ trials, but not animals did 3, we thus conclude that both phenomena depend on the amount of training 3.

## Discussion

Though error-based learning models are well accepted in mammals, there was no evidence of overexpectation, a phenomenon supporting error-based learning models, other than mammals and avians^14,16,17^. The aim of our study was to determine whether overexpectation and its spontaneous recovery could be observed in crickets. We independently trained crickets with two different stimuli (O+ and P+) and then presented the stimuli in compound followed by the same amount of reinforcer (OP+). Crickets that received this treatment responded less to one of the elements of the compound (O) than did crickets in the control group, which had not been conditioned to the compound after elemental conditioning training. The same pattern of results was obtained when we used a different control condition in which crickets were trained with a compound after elemental training. For such a group, the compound consisted of a previously trained odour and a new visual pattern (OP_2_+ : Fig. 2, 3, 4). Our study is, to the best of our knowledge, the first one to show overexpectation in an invertebrate species.

The criteria of overexpectation should be discussed. In overexpectation, separate reinforcement of two stimuli (A and B) followed by reinforcement of the AB compound typically results in a decrease in responding to A. Some practical ways to show the decrease were proposed. For example, Lattal and Nakajima^13^ used a control group that did not receive the compound treatment and the difference between that group and the overexpectation group was presented as evidence of overexpectation. Rescorla^15^ reported overexpectation when, after compound conditioning, CR to A and B decreases relative both to their previous level and to that of similar stimuli not trained in compound. In the latter one, not only the between group comparison but also a within-group comparison was conducted. In our experiments, we found a significant difference between overexpectation and control groups in the three experiments. The preference in the overexpectation group after training was not significantly higher than that before training in experiments 1 (Fig. 2). On the contrary, the preference after 4 OP+ trials in the overexpectation group in experiments 2 and 3 was significantly higher than that before training (Figs. 3 and 4). The latter seems to be natural because the error-based learning models predict slightly but effective learning. For example, the Rescorla-Wagner model predicts that associative strength for A to be less than 1 but more than 0 after overexpectation training (Table 2). The odour pairs used in experiments 2 and 3 are more sensitive, as they enhance learning, than the ones used in experiment 1, thus resulting in a statistically different result. We did not test the preference after O+ and P+ trials before performing OP+ trials, thus we did not see the decrease of preference for O after OP+ in within-group comparison. Based on these results, we concluded that overexpectation occurred in crickets.

In experiments 2 and 3 (Figs. 3 and 4), crickets in the overexpectation group with 4 OP+ trials showed a significantly higher preference for the trained odour in the test 1 day after training than in the test 25 min after training. Such an increase in the conditioned response with the passage of time is known as spontaneous recovery from overexpectation^15^. The results obtained for crickets in the control condition indicate that overexpectation and its spontaneous recovery do not occur when a different stimulus to the one used during the preceding elemental conditioning is used in compound conditioning (control 2).

Three major error-based leaning theories are not consistent with our results as they cannot account for spontaneous recovery from overexpectation, at least in their original forms^19^. When we apply the Rescorla and Wagner model^2^ to our experiment, we can see how, during compound conditioning, the sum of the rewards that are predicted by the odour and the visual pattern (V_Σ_) is of higher value than the reward that is actually received (λ). This results in a negative prediction error that leads to a decreased response: overexpectation (Table 2). However, the model does not predict the spontaneous recovery of the response because it does not include a parameter related to time. According to the attentional theory proposed by Mackintosh^3^, the associative strength gained by the odour (Vo) in O + training should not be higher than the maximum associative strength for the odour and the water reward (λ). Thus, λ-Vo cannot be negative. The learning parameter α, which represents the attention given to a stimulus, is > 0, i. e., α(λ − Vo) is positive, so it predicts excitatory learning during compound conditioning, contrary to our results. The Pearce and Hall model^4^ does not match our results either. In the equation, salience (S), reflecting the intensity of the stimulus, should be positive. The value of α, corresponding to the attentional process as described above, is an absolute value, and λ should be positive as it is appetitive conditioning. Thus, the model predicts excitatory learning in compound conditioning, which is not in agreement with our results.

Rescorla^15^ proposed that overexpectation is analogous to extinction, a process in which repeated presentations of a conditioned stimulus without reinforcement results in a decrease of the conditioned response and a ubiquitous phenomenon widely observed in vertebrates and invertebrates. According to the proposal, spontaneous recovery from extinction or from overexpectation indicates that the excitatory associations formed during initial conditioning remain during extinction or overexpectation training. In addition, it could be argued that when the CS is paired with no US or with an unexpectedly weak US, a new inhibitory association for the CS is established. Whether the animal retrieves the memory of the initial conditioning or of the inhibitory association would depend on the context, which includes the passage of time^20,21^. This view would be helpful to interpret our results. At first, crickets would acquire an excitatory association in O+ training and an inhibitory association during OP+ training. Contrary to the prediction from most conventional error-correction learning theories, the SOP model predicts such acquisition of independent excitatory and inhibitory associations in overexpectation^22^. Then, the inhibitory association that crickets acquired would decay faster than the excitatory association. Thus, preference for O at 1 day after training would be higher than when tested immediately after training, as in experiment 2 and 3. We should note that such a decay is not predicted by the error-correction learning models. According to these views, there is a need to modify conventional error-correction learning theories so that they can account for spontaneous recovery from extinction or overexpectation. Some of such refinements have been proposed for mammals^19^.

In insects, spontaneous recovery from extinction has been reported after conditioning an odour CS with sucrose in the honeybee *Apis mellifera*^23^. In bees, it has been proposed that reconsolidation of the original excitatory memory underlies spontaneous recovery^24^. Reconsolidation is the process in which previously consolidated memories become labile again through reactivation of the memory trace and are then consolidated again. Whether spontaneous recovery form overexpectation in crickets can be accounted for by the same mechanisms needs to be investigated.

The neural mechanisms that lead to a reduction of the CR due to overexpectation and to the subsequent recovery of the CR are still to be investigated. In mammals, a brief inhibition of dopaminergic neurons in the midbrain during Pavlovian training mimics the reduced CR that is observed in an overexpectation preparation, which suggests that midbrain dopamine neurons convey negative prediction error signals during conditioning^25^. However, the neural basis of spontaneous recovery from extinction or overexpectation remains unknown. In the case of crickets, our previous studies suggest that octopamine and dopamine neurons convey positive prediction error signals for appetitive and aversive rewards, respectively^9,10^. Investigation of the activity of these neurons during overexpectation training is a future subject in crickets.

In conclusion, the results of this study show that conventional error correction learning models account for some essential features of Pavlovian conditioning in crickets but not for all of them. Hence, more effort is needed to better elucidate associative processes underlying overexpectation, its spontaneous recovery and their neural mechanisms in crickets. Many experimental procedures, such as pharmacology, RNAi and genome editing with the CRISPR/cas9 system, are available for investigating the neural processes underlying Pavlovian conditioning in crickets^26–30^. Crickets may provide a very useful model to clarify the neural mechanisms of overexpectation and its spontaneous recovery.

## Materials and methods

### Experimental animals

Crickets, *Gryllus bimaculatus*, were kept in laboratories more than 5 years in Tokyo Medical and Dental University and Hokkaido University. They were reared in a 12 h: 12 h light: dark cycle at 27 ± 2 °C and were fed a diet of insect pellets and water ad libitum. At 1 week after the imaginal molt, male crickets were collected randomly and were placed individually in beakers and deprived of drinking water for 3 days to enhance their motivation to search for water. Experiments are demonstrated under permission by Tokyo Medical and Dental University and Hokkaido University.

### Olfactory and visual conditioning procedure

Conditioning was conducted according to procedures previously described^9^. Syringes were used for presenting the CSs and the US to the crickets during conditioning. They contained water as appetitive US. For olfactory conditioning, a small filter paper soaked with odour essence was attached to the needle of each syringe to present the olfactory CS. For visual conditioning, a pattern was attached to the needle. For compound conditioning, both stimuli were simultaneously performed. In each conditioning trial, an odour and/or a visual pattern was approached to the head of the cricket and held for 3 s. After that time, a drop of water was given to the mouth of the animal.

Olfactory conditioning consisted of 3 trials in which an odour (apple, banana, or peppermint (referred to as mint)) was paired with water (O+ trials). Visual conditioning consisted of 4 trials in which a white-centre and black-surround pattern (WB pattern) was paired with water (P+ trials). Compound conditioning consisted of 4 trials in which the odour used during the first part of the training and the visual pattern were simultaneously presented and reinforced with water (OP+ trials). These numbers of conditioning trials are sufficient to achieve association^9,26,27^. Additionally, in Experiment 3, a different visual pattern was presented in compound with the odour. In this case, a black-centre and white-surrounding pattern (BW pattern) was used as a control, which cricket can discriminate from WB pattern^27^. The intervals between the conditioning trials (inter-trial intervals, ITIs) were 5 min.

### Preference test

The procedure of the test was described previously^9^. The floor of the test chamber had two holes that connected the chamber with two cylindrical containers. The containers contained a filter paper soaked with odour essence and were covered with a fine gauze net. Three containers were mounted on a rotative holder, and two of the three containers could be located simultaneously beneath the holes of the test chamber. We presented odour pairs on the chamber to record the relative odour preference. In experiments in which apple or banana odours was used as the CS, both were presented in the chamber, since crickets´ innate preference for either of them is almost equal. In experiments in which mint was used as the CS, we presented mint and vanilla in the chamber. The rationale for using vanilla and mint is that crickets innately prefer vanilla to mint odour; thus, appetitive conditioning to mint odour can be detected more clearly than when using the apple/banana odour pair.

Crickets were individually placed in a waiting chamber within the experimental setup for four minutes so they could acclimatise to it. Then the cricket was gently pushed to the entrance of the test chamber and the test started when it entered the chamber. Two min after the test had started, the relative positions of the odour sources were changed by rotating the container holder. The preference test lasted for 4 min. We considered that the cricket visited an odour source when it probed the top net with its mouth or palpi. The time that the cricket spent at each odour source was recorded cumulatively for each second. If the total visiting time of a cricket to the odour sources was less than 10 s, we considered that the animal was less motivated, possibly due to a poor physical condition, and the data were discarded. About 20% of the animals were rejected in each experiment. Experiments 1 and 2 are demonstrated by KT. In experiment 3, conditioning and preference tests are demonstrated independently by KT and YM in the blind condition.

### Statistics

The relative preference for the conditioned odour was determined as the proportion of time that the crickets spent visiting the conditioned odour compared to the total amount of time that they spent visiting the two odours. We used a generalized linear mixed model (GLMM) to evaluate the relative odour preference with a binomial distribution of the relative preference, determined by the search time data sampled for each second, and a logit link function as described previously^10^. We included test condition (test), training procedure (training), number of trials (trials), and/or their interaction as fixed effects in the GLMM, with the training and test terms being categorical variables, and trials as quantitative variables. Identifier (ID) of each cricket was used as a random effect, allowing for the random intercept. Post-hoc analyses were executed with the same function and distribution. P-value was adjusted by the Holm method. For statistical analysis, we used the software R (ver 3. 5. 1) and the packages lme4 (ver. 1.1.12) and emmeans (ver 1. 6. 1)^31–33^.

## Supplementary Information

Supplementary Information.
