# Supplementary material for: Spontaneous recovery from overexpectation in an insect
Source: Sci Rep. 2022 Jun 14;12:9827. doi: 10.1038/s41598-022-13800-2 (PMC9198028; doi:10.1038/s41598-022-13800-2)
Supplement: Supplementary file 1 — Supplementary Information. [file 41598_2022_13800_MOESM1_ESM.pdf]

Spontaneous recovery from overexpectation in an insect

Kanta Terao[1, 2*], Yukihisa Matsumoto[1], Beatriz Álvarez [3], Makoto Mizunami[4*]

[1]College of Liberal Arts and Sciences, Tokyo Medical and Dental University, Ichikawa 272-0827, Japan

[2]Research Institute for Letters, Arts and Sciences, Waseda University, 1-24-1 Toyama, Shinjuku, 162-8644 Japan

[3]Facultad de Ciencias de la Salud, Universidad Pública de Navarra, 31006, Spain

[4]Faculty of Science, Hokkaido University, Sapporo 060-0810, Japan

*Corresponding authors: tkanta.las@tmd.ac.jp (KT), mizunami@sci.hokudai.ac.jp (MM)

Supplemental Table 1. Summary of the statistics for experiment 1 (Fig. 2).

Fixed effects	Estimate	Standard error	Z ratio	P value
Intercept	-0.0913	0.130	-0.704	0.481
Test	-0.0982	0.187	-0.524	0.600
Training	0.123	0.0950	1.29	0.196
Interaction (immediate x control)	0.690	0.139	4.96	$7.07 * 10^{-7}$

Effects of the test situation (pre or immediate test), training procedure (overexpectation or control procedure) and interaction between them on the relative preference for the conditioned odour were evaluated by using a GLMM. The estimate for the intercept represents the estimate for the overexpectation group in the pre-test.

Supplemental Table 2. Summary of the statistics for all data in experiment 2 (Fig. 3).

Fixed effects	Estimate	Standard error	Z ratio	P value
Intercept	-1.03	0.138	-7.48	$7.51 * 10^{-14}$
Test (immediate)	0.240	0.106	2.26	0.0239
Test (1 day after)	0.567	0.111	5.12	$2.99 * 10^{-7}$
Training (control)	0.139	0.149	0.929	0.353
Training (control 2)	0.0539	0.199	0.270	0.787
Interaction (immediate x control)	1.40	0.157	8.93	$4.42 * 10^{-19}$
Interaction (1 day after x control)	0.978	0.162	6.05	$1.48 * 10^{-9}$
Interaction (immediate x control 2)	1.45	0.157	9.20	$3.46 * 10^{-20}$
Interaction (1 day after x control 2)	1.08	0.163	6.59	$4.25* 10^{-11}$

Effects of the test situation, training procedure and interaction between them on the relative preference for the conditioned odour were evaluated. The estimate for the intercept represents the estimate for the overexpectation group in the pre-test.

Supplemental Table 3. Summary of the post-hoc analyses for experiment 2 (Fig. 3)

a. analysis for the results in pre-test

Data1	Data2	Estimate	Standard error	Z.ratio	P value
overexpectation	control	-0.139	0.149	-0.929	1
overexpectation	control 2	-0.0539	0.199	-0.270	1
control	control 2	0.0848	0.200	0.423	1

b. analysis of the results in the immediate test

Data1	Data2	Estimate	Standard error	Z.ratio	P value
overexpectation	control	-1.54	0.142	-10. 9	$5.61* 10^{-27}$
overexpectation	control 2	-1.50	0.183	-8.22	$4.03 * 10^{-16}$
control	control 2	0.0415	0.194	0.214	0.830

c. analysis of the results in the 1-day-after test

Data1	Data2	Estimate	Standard error	Z.ratio	P value
overexpectation	control	-1.12	0.148	-7.52	$1.60 *10^{-13}$
overexpectation	control 2	-1.13	0.189	-6.00	$3.83 *10^{-9}$
control	control 2	-0.0155	0.198	-0.0785	0.937

d. analysis of the results in the overexpectation group

Data1	Data2	Estimate	Standard error	Z.ratio	P value
pre	Immediate	-0.240	0.106	-2.26	0.0239

pre	1day	-0.567	0.111	-5.12	$8.97 * 10^{-7}$
immediate	1day	-0.327	0.0931	-3.51	0.000897

e. analysis of the results in the control group

Data1	Data2	Estimate	Standard error	Z.ratio	P value
pre	Immediate	-1.64	0.116	-14.2	$2.28 * 10^{-45}$
pre	1day	-1.54	0.118	-13.1	$4.84 * 10^{-39}$
immediate	1day	0.0997	0.112	0.892	0.372

f. analysis of the results in the control 2 group

Data1	Data2	Estimate	Standard error	Z.ratio	P value
pre	Immediate	-1.69	0.116	-14.6	$1.58 * 10^{-47}$
pre	1day	-1.64	0.120	-13.7	$3.37 * 10^{-42}$
immediate	1day	0.0427	0.101	0.422	0.673

Effects of the test situation or training procedure on relative preference for the conditioned odour were evaluated by comparing each set of data.

Supplemental Table 4. Summary of the statistics for all data in experiment 3 (Fig. 4).

Fixed effects	Estimate	Standard error	Z value	P value
Intercept	-1.12	0.188	-5.96	$2.47*10^{-9}$
Test (immediate)	1.94	0.134	14.4	$4.00*10^{-47}$
Test (1 day after)	1.91	0.139	13.8	$4.04*10^{-43}$
Trials	0.0688	0.0668	1.03	0.303
Interaction (immediate x trials)	-0.455	0.0458	-9.95	$2.56*10^{-23}$
Interaction (1 day after x trials)	-0.376	0.0473	-7.95	$1.94*10^{-15}$

Effects of the test situation, amount of training trials and interaction between them on the relative preference for the conditioned odour were evaluated. The estimate for the intercept represents the estimate in the pre-test for trials 0.

Supplemental Table 5. Summary of the post-hoc analyses for experiment 3 (Fig. 4)

a. analysis of the results in the pre-test

Data1	Data2	Estimate	Standard error	Z.ratio	P value
trial 0	trial 1	0.188	0.330	0.570	1
trial 0	trial 2	-0.0717	0.407	-0.176	1
trial 0	trial 3	-0.197	0.325	-0.606	1
trial 0	trial 4	-0.118	0.302	-0.392	1
trial 1	trial 2	-0.260	0.385	-0.674	1
trial 1	trial 3	-0.385	0.296	-1.30	1
trial 1	trial 4	-0.306	0.271	-1.13	1
trial 2	trial 3	-0.125	0.380	-0.329	1
trial 2	trial 4	-0.0467	0.361	-0.129	1
trial 3	trial 4	0.0785	0.265	0.296	1

b. analysis of the results in the immediate test

Data1	Data2	Estimate	Standard error	Z.ratio	P value
trial 0	trial 1	-0.299	0.309	-0.969	0.998
trial 0	trial 2	0.136	0.377	0.360	1
trial 0	trial 3	1.13	0.310	3.63	0.00198
trial 0	trial 4	1.12	0.284	3.96	0.000609
trial 1	trial 2	0.435	0.353	1.23	0.875
trial 1	trial 3	1.43	0.282	5.06	$3.73*10^{-6}$
trial 1	trial 4	1.42	0.252	5.64	$1.73*10^{-7}$
trial 2	trial 3	0.991	0.354	2.79	0.0260

trial 2	trial 4	0.988	0.332	2.98	0.0174
trial 3	trial 4	-0.00262	0.254	-0.0103	1

c. analysis of the results in the 1-day-after test

Data1	Data2	Estimate	Standard error	Z.ratio	P value
trial 0	trial 1	-0.189	0.315	-0.601	1
trial 0	trial 2	0.0775	0.397	0.195	1
trial 0	trial 3	1.078	0.314	3.44	0.00473
trial 0	trial 4	0.873	0.291	3.00	0.0187
trial 1	trial 2	0.267	0.372	0.717	1
trial 1	trial 3	1.27	0.281	4.50	$6.75*10^{-5}$
trial 1	trial 4	1.06	0.256	4.15	0.000293
trial 2	trial 3	1.00	0.371	2.70	0.0418
trial 2	trial 4	0.796	0.352	2.26	0.118
trial 3	trial 4	-0.204	0.254	-0.804	1

d. analysis of the results in 0 trials

Data1	Data2	Estimate	Standard error	Z.ratio	P value
pre	Immediate	-1.46	0.188	-7.77	$1.52*10^{-14}$
pre	1day	-1.56	0.196	-7.96	$5.21*10^{-15}$
immediate	1day	-0.100	0.169	-0.591	0.554

e. analysis of the results in 1 trials

Data1	Data2	Estimate	Standard	Z.ratio	P value

			error		
pre	Immediate	-1.95	0.155	-12.6	$7.86*10^{-36}$
pre	1day	-1.94	0.157	-12.3	$1.79*10^{-34}$
immediate	1day	0.0102	0.134	0.0760	0.939

f. analysis of the results in 2 trials

Data1	Data2	Estimate	Standard error	Z.ratio	P value
pre	Immediate	-1.25	0.213	-5.86	$1.36*10^{-8}$
pre	1day	-1.41	0.246	-5.74	$1.89*10^{-8}$
immediate	1day	-0.158	0.210	-0.751	0.453

g. analysis of the results in 3 trials

Data1	Data2	Estimate	Standard error	Z.ratio	P value
pre	Immediate	-0.136	0.145	-0.938	0.546
pre	1day	-0.284	0.138	-2.06	0.119
immediate	1day	-0.148	0.135	-1.10	0.546

h. analysis of the results in 4 trials

Data1	Data2	Estimate	Standard error	Z.ratio	P value
pre	Immediate	-0.217	0.107	-2.03	0.0421
pre	1day	-0.567	0.111	-5.11	$9.78*10^{-7}$
immediate	1day	-0.350	0.0940	-3.73	0.00039

Effects of the test or number of training trials on the relative preference for the conditioned odour were evaluated by comparing each set of data.
